# Supplementary material for: Metagenomic profiles of archaea and bacteria within thermal and geochemical gradients of the Guaymas Basin deep subsurface
Source: Nat Commun. 2023 Nov 27;14:7768. doi: 10.1038/s41467-023-43296-x (PMC10681998; doi:10.1038/s41467-023-43296-x)
Supplement: Supplementary file 1 — Supplementary Information [file 41467_2023_43296_MOESM1_ESM.pdf]

Metagenomic profiles of Archaea and Bacteria within Thermal and Geochemical Gradients of the Guaymas Basin Deep Subsurface

Paraskevi Mara, David Geller-McGrath, Virginia Edgcomb, David Beaudoin, Yuki Morono, Andreas Teske

Supplementary Note

In this Supplementary Note we provide an extended overview on the genomic background of bacterial and archaeal lineages that dominate the Guaymas subsurface metagenome and MAG surveys, and we describe certain genes involved in carbon and iron cycling that we recovered on most of our MAGs. We focus our discussion on Chloroflexota (carbon fixation, DMSO reduction), Thermoproteota (acetogenesis and methane cycling), Acidobacteriota (nitrogen fixation), Desulfobacterota (sulfate and iron reduction), Aerophobota, and the White Oak River group 3 (WOR-3) (diverse heterotrophic capabilities, and CRISPR genes). We introduce further metagenomic evidence for iron reduction and oxidation, and carbon monoxide oxidation. We include a section that examines the application of bioinformatic tool "MetaPathPredict" (Geller-McGrath et al. 2022) and its insights into the metabolic potential of MAGs assigned to less dominant phyla (Zixibacteria and Cloacimonadetes) in the Guaymas Basin subsurface. Finally, at end of this Supplementary Note, we devote a section that explains the processing and the rationale behind the control samples used in this study.

Overview on the genomic background of subsurface bacterial and archaeal phyla in Guaymas Basin

Chloroflexota. Chloroflexota are abundant in the hadal ocean (Liu et al., 2022a), in marine subsurface sediments (Vuillemin et al., 2020, Fincker et al. 2020,) and hydrothermal settings (Fullerton and Moyer, 2016, Dombrowski et al., 2018; Reysenbach et al., 2020). Diverse thermophilic Chloroflexota lineages have also been enriched and isolated from hot springs (Dodsworth et al., 2014; Palmer et al., 2023).

Genes detected in our Chloroflexota MAGs are associated with fatty acid degradation, ornithine biosynthesis and the methionine salvage pathway that produces methionine by recycling sulfur-bearing metabolites (Sekowska et al., 2004). Ornithine biosynthesis was also evident in MAGs affiliated with other bacterial and archaeal phyla, including the Hadarchaeota. Genes for enzymes involved in ornithine synthesis (e.g., ornithine decarboxylase) have been documented previously in deep biosphere samples (Orsi et al., 2013), indicating that this capability may be widely utilized by subsurface microbiota (Hernández et al., 2021).

The majority of Chloroflexota genomes contained marker genes associated with the oxidation of formate (*fdhA*, *fdhB*, *fdoG*, *fdoH*; McGonigle et al., 2020) and carbon monoxide (*coxL*, *coxM*, *coxS*; Islam et al., 2019). Their occurrence coincides with increased carbon monoxide concentrations (83-665 nM) between 0.8-60 mbsf (Supplementary Dataset 1). Hydrogen utilization is suggested by Ni-Fe hydrogenases and key genes of acetate/acetyl-CoA production (*pta*/*ack*, *acdA*) in Chloroflexota MAGs (Supplementary Datasets 5, 6). Hydrogen concentrations associated with our metagenomic samples ranged between 23-84 nM (Supplementary Dataset 1) indicative of active hydrogen cycling (Lin et al., 2012). Genes associated with the Wood-Ljungdahl pathway (WL; *cdhD*, *cdhE*, *cooS*) were present in 17/23 Chloroflexota MAGs, while

ATP-citrate lyase (*aclA*), associated with the reductive TCA cycle (rTCA), was present in one MAG from the VGOG01 order. The *mmoB* gene associated with methane oxidation (Supplementary Dataset 7) was identified in two Chloroflexota MAGs from the orders Promineofilales (Speirs et al., 2019) and E44-bin15, associated with petroleum seepage in marine sediments (Dong et al. 2019). Previous DNA-SIP experiments identified members of the Chloroflexota as putative methane oxidizers and detected methane monooxygenases (*mmoX* or *pmoB)* in Chloroflexota genomes (Altshuler et al., 2022). We caution that the identified *mmoB* has a regulatory role in methane oxidation, while the catalytic activity is encoded by the *mmoH* gene in the *mmo* operon (Sirajuddin and Rosenzweig, 2015).

Genes involved in sulfate and sulfur assimilation for amino acid synthesis (e.g., cysteine; *cysN*, *sat*, *cysD*) and in sulfur oxidation *(sdo*, *dsrH)* were present in all Chloroflexota MAGs, however, none of our Chloroflexota MAGs encoded complete pathways for sulfur oxidation. Evidence for dimethyl sulfoxide (DMSO) utilization was suggested by the presence of *dmsA* and/or *dmsB* (involved in DMSO reduction) in five of our MAGs (Supplementary Datasets 5, 6). DMSO is abundant in deep sea ecosystems and was suggested to be an electron acceptor for microbes that survive in deep-sea extreme conditions (Xiong et al., 2016). Genes for denitrification, (e.g., *nirB* and *nirD*) were encoded in one Chloroflexota MAG. Finally, genes for transport of tungstate were identified in 4 Chloroflexota MAGs, and transporters of molybdenum/tungsten in 10 MAGs (Supplementary Datasets 5, 6). Tungsten is found abundantly in hydrothermal ecosystems (Kishida et al., 2004), and serves as a redox catalyst in metalloenzymes of thermophilic archaea inhabiting hydrothermal vents (Kletzin and Adams, 1996) and hot springs (Buessecker et al., 2022). Evidence of putative arsenate biomineralization for detoxification (e.g., *arsC*, *arsM* genes) or energy gain via arsenotrophy (*arrA* gene; Saunders

et al., 2019) was present in three MAGs. Sedimentary arsenic input would explain the elevated concentrations of arsenic in Guaymas Basin hydrothermal fluids (up to 1 µmol) that exceed those at other hydrothermal vent sites (Von Damm et al., 1985). Five Chloroflexota MAGs contained CRISPR/Cas genes involved in genome editing (Supplementary Datasets 5, 6).

Thermoproteota. Bathyarchaeia recycle hydrogen and CO_2 from fermentation using the WL pathway (He et al., 2016). Eight out of 11 of our Bathyarchaeia MAGs contained genes involved in the WL pathway. In addition, we detected the marker gene for the formaldehyde activating enzyme (*fae*) in 5 MAGs affiliated with 40CM-2-53-6, B26-1 and TCS64 orders. *Fae* condenses formaldehyde and tetrahydromethanopterin to form methylene-tetrahydromethanopterin that can be reduced and utilized in the WL pathway (Timmers et al., 2017; Vorholt et al., 2000). Six Bathyarchaeia MAGs encoded the *mer* gene (*ffdA* synonym, to avoid confusion with the *mer* operon for mercury reduction) for the reduction of methylene-tetrahydromethanopterin in the WL pathway. Various Bathyarchaeia sub-lineages have been reported to encode genes for anaerobic oxidation of methane/alkane compounds (*mcr*/*acr* complex) (Evans et al., 2015, Evans et al., 2019; Qi et al., 2021; Vanwonterghem et al., 2016). We detected the methane/alkane oxidation marker genes *fwd*, *ftr*, *mtd*, *mch*, and *mtr* in all Bathyarchaeia MAGs. However, the *acr*/*mcr* genes encoding the methyl/alkyl-coenzyme M complex were absent, indicating loss of the *mcr*/*acr* operon as described previously for Bathyarchaeia. Nine out of 11 Bathyarchaeia MAGs also encoded genes for acetate formation (*acdA*, *ack*, *pta, acs)* which could be utilized to couple methylotrophy with acetogenesis, as has been described previously in Bathyarchaeia MAGs from deep sediments (Farag et al., 2020; He et al., 2016).

Marker genes for other specific metabolic capacities in our Bathyarchaeota MAGs included genes for fermentation (*porA*), hydrogen cycling (Ni-Fe hydrogenases) and genes involved in the anaerobic degradation of benzoate (*bcrA, bcrB, bcrD*; Kung et al., 2009). The *bcr* genes were also observed in Chloroflexota (5 MAGs), Zixibacteria (1 MAG) and Desulfobacterota (4 MAGs); the latter group can be enriched in Guaymas Basin sediments on benzoate, under sulfate-reducing conditions (Edgcomb et al., 2022).

Acidobacteriota. Members of the highly diverse heterotrophic phylum Acidobacteriota occur in a wide range of freshwater and marine seafloor environments and can utilize oxygen or other electron acceptors (e.g., nitrate, nitrite, sulfate) for respiration (Flieder et al., 2021 and references therein). We detected eight Acidobacteriota MAGs annotated to the orders of Aminicenantales (7 MAGs), and Acidoferrales (1 MAG). Aminicenantales MAGs were previously recovered from surficial Guaymas Basin sediments (Dombrowski et al., 2018), and in this study they were found at all sites between 0.8-60 meters below sea floor (mbsf). The Acidoferrales MAG was detected only below 61.6 mbsf at sites U1545B and U1548B. The overall metabolic potential of Guaymas subsurface Acidobacteriota MAGs is summarized in Supplementary Datasets 5 and 6.

We observed that almost all Acidobacteriota MAGs encoded the NtrY-NtrX two-component regulatory system, a redox sensor system widely distributed in Proteobacteria which regulates denitrification and nitrogen fixation genes, and senses nitrogen levels under nitrogen limitation (Pawlowski et al., 1991; DelVecchio et al., 2002; Bonato et al., 2016). Six of our eight Acidobacteriota MAGs contained at least one gene of the *nif* operon (e.g., *nifU, nifB, nifH*) which suggests putative nitrogen fixation in our subsurface samples. This is similar to previous reports of deep-sea sediment Acidobacteriota that encode *nifH* in their genomes (Kapili et al., 2020). Two

Acidobacteriota MAGs contained CRISPR/Cas genes involved in genome editing (Supplementary Dataset 5).

Desulfobacterota. Desulfobacterota include primarily heterotrophic sulfate reducers and syntrophic sulfate-reducing lineages that couple sulfate reduction with methane and short-chain alkane oxidation by anaerobic methane oxidizers (ANME archaea). These bacteria are widespread in Guaymas Basin sediments and other hydrothermal and cold seep sites (Knittel and Boetius, 2009; Murphy et al., 2021; Speth et al., 2022; Wegener et al., 2022; Zhou et al., 2022). Seven Desulfobacterota MAGs, belonging to the Desulfobacterales, Desulfatiglandales, and WTBG01 and WVXP0 orders were recovered primarily from shallow sulfate-rich cool sediments of all sites (0.8-15 mbsf, at or above the SMTZ with temperatures 2-20°C) (Supplementary Figure 4). Two Desulfobacterales MAGs contained the *dsr* operon (e.g., *dsrB/J/K/D*) involved in dissimilatory sulfate reduction (Venceslau et al., 2014). One MAG annotated to the WTBG01 order (found in freshwater anoxic sulfidic sediments; Murphy et al., 2021) encoded the sulfate adenylyltransferase gene (*sat*) associated primarily with sulfur assimilation. Marker genes of DMSO reduction and/or sulfur assimilation were also detected in all Desulfobacterota MAGs. One Desulfobacterota MAG contained CRISPR/Cas genes involved in genome editing (Supplementary Dataset 5).

The potential for iron reduction was evidenced in all Desulfobacterota MAGs by the presence of *mtrA*, *mtoA* (Garber et al., 2020) and *eetB* genes, suggesting an extracellular electron transfer mechanism. In addition, *DFE* genes encoding multiheme cytochromes (e.g., *DFE_0449, DFE_0461, DFE_0451*) were found in three of our MAGs annotated to Desulfobacterota and in five MAGs annotated to Aminicenantales (Acidobacteriota). *DFE* genes are involved in iron oxidation and were originally documented in the genome of *Desulfovibrio ferrophilus* strain IS5

(Deng and Okamoto, 2018). They encode cytochromes and β-propeller proteins, which can function as electron carriers and leader peptides in extracellular electron transfer (Chatterjee et al., 2021; Deng and Okamoto, 2018). At depths where Desulfobacterota MAGs were detected, dissolved porewater iron ranged in concentration from < 1 µM to greater than 4 µM (Supplementary Dataset 1), indicating possibly active iron cycling with little accumulation.

Aerophobota and White Oak River group 3 (WOR-3). The phylum Aerophobota is widely distributed in deep-sea sediments, and includes fermentative thermophiles and hyperthermophiles affiliated with hydrocarbon-rich environments such sediments from the Pescadero Basin (Speth et al., 2022), and methane hydrate-bearing sediments (Liu et al., 2022b). We recovered five Aerophobota MAGs (order Aerophobiales) from the deep subsurface at depths below 100 mbsf, when temperatures did not exceed 40°C. This suggests that the distribution of Aerophobota appears to be constrained by thermal limits rather than by depth. Guaymas Aerophobota MAGs encoded marker genes for acetate/acetyl-CoA production (*acdA*, *ack*, *pta*) (Dong et al., 2019), fermentation (*porA*) and degradation of polysaccharides (e.g., cellulose, chitin). Two Aerophobota MAGs contained CRISPR/Cas and CRISPR/Csm genes that comprise adaptive defense systems against infectious agents in prokaryotes (Colognori et al., 2023).

Members of the bacterial WOR-3 candidate phylum were originally described from shallow estuarine sediments (Baker et al., 2015) and hydrothermal Guaymas Basin sediments (Dombrowski et al., 2017). Five of our six WOR-3 MAGs were recovered from depths at 0.8-26.9 mbsf; in contrast, a WOR-3 MAG annotated to UBA3073 (order-level) was recovered primarily from 112.5 and 154.2 mbsf at site U1545 (up to ~45°C) (Figure 2). Overall, our WOR-3 MAGs encoded various peptidases, as well as genes for H_2 cycling (Ni-Fe hydrogenase genes) and

putative chitin degradation (endo-acting chitinase genes). While these results match previous findings (Baker et al., 2015), the Guaymas subsurface WOR-3 MAGs also contain genes for fermentation and acetate production (*acs, acdA*, *ack, porA*), and marker genes for endohemicellulases and amylolytic enzymes that can degrade other polysaccharides aside from chitin. One WOR-3 MAG encoded CRISPR/Cas genes.

Iron reduction and oxidation. Iron reduction is a known capability for Bacteria and Archaea associated with marine benthic sediments (Flieder et al., 2021; Jiang et al., 2019). Iron (II and III) concentrations associated with metagenomic samples containing MAGs that encoded genes affiliated with iron metabolism ranged from nanomolar, up to 11.8 µM (Supplementary Dataset 1). Marker genes involved in dissimilatory iron reduction (e.g., *dmkA*, *dmkB*, *eetA*, *eetB*, *fmnA*, *fmnB*, *pplA*, *ndh2*; Garber et al., 2020) with the potential for extracellular electron transfer (EET) were identified in 86/89 MAGs from all recovered phyla (Supplementary Datasets 5, 6), and were only missing from one Thermoproteota, one WOR-3, and one Aenigmatarchaeota MAG. These genes participate in EET from the cell towards the surrounding environment (Light et al., 2018; Shi et al., 2016). Based on laboratory experiments, EET is suggested to enhance iron bioavailability and iron uptake in anaerobes, and to act as a redox mechanism that may contribute to the proton motive force (Jeuken et al., 2020).

Eight out of 23 Chloroflexota, 2/11 Thermoproteota, 5/8 Acidobacteriota, 3/7 Desulfobacterota, and 1/6 WOR-3 MAGs also encoded components of the *DFE_0448-0451* and *DFE_0461-0465* operon genes (Supplementary Datasets 5, 6) homologous to multiheme cytochrome systems first identified in *Desulfovibrio ferrophilus* (Deng et al., 2018). According to the *D. ferrophilus* model, electrons from an external iron source move along extracellular and

membrane-spanning multiheme cytochromes from the outer membrane to the periplasm of the cell and finally are passed to a terminal electron acceptor (Deng et al., 2018). Putative terminal electron acceptors for this process in MAGs containing *DFE* components include the sulfur cycle intermediates sulfite (based on the presence of sulfite reductase *asrA* and *asrB*), tetrathionate (from the detection of tetrathionate reductase gene *ttrB*), and thiosulfate (due to the annotation of thiosulfate reductase *phsA/B* genes; Supplementary Datasets 5, 6). The capacity for polysulfide reduction was also detected based on the presence of polysulfide reductase (*psrA*) in four Acidobacteriota MAGs containing *DFE* multiheme cytochrome components. Electron acceptors from nitrogen cycle intermediates included nitrite (*nasD*) and nitrate (*napA*, *narB*).

Carbon monoxide oxidation. Hydrothermal environments often contain carbon monoxide (CO), which can be produced by the breakdown of organic matter or generated by certain anaerobic microorganisms (Kochetkova et al., 2011; Sokolova et al., 2009). The potential for CO oxidation, is an energetically favorable anaerobic reaction prevalent in subsurface bacterial and archaeal MAGs (Baker et al., 2016; Magnabosco et al., 2016). Nine out of 23 Chloroflexota MAGs, 3/11 Thermoproteota, 7/8 Acidobacteriota, 3/7 Desulfobacterota, 2/5 Aerophobota, and one Hadarchaeota MAGs contained the genes *coxM* and *coxS* encoding CO dehydrogenase subunits (Supplementary Datasets 5, 6). Genes for catalytic nickel-containing CO dehydrogenase (*cooS*) and/or its iron-sulfur subunits (*cooF*) were further identified in 9/23 Chloroflexota, 1/11 Thermoproteota, 1/8 Acidobacteriota, 3/7 Desulfobacterota, and 2/5 Aerophobota MAGs. The presence of these genes suggests the capacity to oxidize CO and H_2O to CO_2 while reducing an electron-carrying cofactor that is accepting H_2. Bacteria and Archaea are also capable of CO oxidation coupled to the anaerobic reduction of sulfur and nitrogen compounds (King, 2006;

Oelgeschläger and Rother, 2008). Potential terminal electron acceptors for our CO-oxidizing MAGs included the sulfur cycle intermediates sulfite (due to the presence of genes *asrA* and *asrB*), tetrathionate (*ttrB*), thiosulfate (*phsA/B*), and polysulfide (*psrA*; Supplementary Datasets 5, 6). CO concentrations associated with metagenomic samples containing MAGs that encoded these genes ranged from 83 to 665 nM (Supplementary Dataset 1).

MetaPathPredict insights into metabolic potential of Zixibacteria and Cloacimonadetes.

Some phyla for which we recovered MAGs (e.g., Zixibacteria and Cloacimonadota) remain poorly described in terms of their metabolic potential. To assess and predict the metabolic potential of some of our less-complete and poorly characterized bacterial MAGs, we applied a new tool "MetaPathPredict" (Geller-McGrath et al., 2022) to our Zixibacteria (n= 4) and Cloacimonadota (n=1) MAGs. MetaPathPredict is a software designed to predict the presence or absence of complete KEGG modules in partially complete bacterial genomes (see below). The tool utilizes machine learning models trained on bacterial gene annotations in the format of KEGG gene orthologs to predict the presence or absence of whole KEGG modules, and it has been designed to handle gene annotations from incomplete bacterial genomes. It is very common in environmental metagenomic studies that reconstructed MAGs will vary in their degree of completeness and contamination. Usually only a fraction of MAGs exceeds > 80% completeness, and it is known that less complete MAGs can result in underestimation of the functional capacity of the genome (Eisenhofer et al., 2023), which can be particularly important for uncultured bacterial taxa. Application of MetaPathPredict to such partially complete MAGs can be useful for providing predictions on whether metabolic pathways are present especially in cases where some key genes are missing. While this tool can sometimes miss predictions of pathways that should be present

based on benchmarking tests with genomic data (Geller-McGrath et al., 2022), it can nonetheless be useful for gaining insights into less-complete MAGs, and also for predicting metabolic capacities of MAGs affiliated with poorly understood taxonomic groups.

Our Zixibacteria MAGs were 61-88.5% complete and our single Cloacimonadota MAG was 75% complete. The results of the MetaPathPredict analysis are described below and presented in Supplementary Dataset 10 which shows: 1) KEGG modules present and complete in our Zixibacteria and Cloacimonadota MAGs that were also predicted by MetaPathPredict 2) KEGG modules absent or incomplete but predicted to be present by MetaPathPredict, and 3) incomplete KEGG modules, also predicted to be absent by MetaPathPredict.

Lineages of Zixibacteria have been documented in various marine and terrestrial subsurface ecosystems, hypersaline settings, and anoxic sediments (Anantharaman et al., 2018; Baker et al., 2015; Castelle et al., 2013; Lin et al., 2012b; Momper et al., 2017; Wong et al., 2020). This taxon is thought to be capable of dissimilatory nitrate and sulfate reduction, and it seems to lack complete carbon fixation pathways (Momper et al., 2017). The 4 Zixibacteria MAGs we recovered were detected at all sampling sites down to 25.8 mbsf and metagenome reads mapped most intensely from shallow/intermediate depths (8.6-16.2 mbsf). Zixibacteria (order MSB-5A5) have the metabolic capacity for oxidation of fatty acids, (e.g., acyl-CoA dehydrogenase) and synthesis of a suite of vitamins (e.g, B6, B1). MetaPathPredict predicted the potential for dissimilatory sulfate reduction in one of our MAGs (order DG-27), which also contained a marker gene for this process (*dsrA*). Two Zixibacteria MAGs (orders DG-27 and UBA10806) were also predicted to encode the potential for dissimilatory nitrate reduction and contained marker genes *napB* and *nrfH*. MetaPathPredict additionally predicted the synthesis of vitamin B7 in two Zixibacteria MAGs, and acetate production via the phosphate acetyltransferase-acetate kinase

(Pta-Ack) pathway in all four MAGs. The Pta-Ack pathway can produce acetate/acetyl-CoA and can participate in carbon fixation by providing acetyl-CoA. Complete acetogenesis via the pta-ack pathway was also confirmed from our genomic data in two MAGs (*ack*, *pta*). MetaPathPredict predicted various transporters (e.g., phosphate, iron and ABC transporters), pathways involved in synthesis of co-factors using amino acids or tRNA reductases (e.g., from glutamate to heme; from tRNA-glutamyl to sideroheme), and the synthesis of various amino acids (e.g., threonine, serine, valine isoleucine). Many of these processes (e.g., biosynthesis of amino acids, iron reduction) were also verified with marker genes. The Zixibacteria MAGs did not contain the potential for carbon fixation.

Cloacimonadota are abundant in anoxic/sulfidic water columns and cold seep brine pools (e.g., Black and Red Sea, respectively; Suominen et al., 2021; Villanueva et al., 2021; Zhang et al., 2016). They are suggested to perform diverse metabolisms including carbon fixation, fermentation, and assimilation of proteins as carbon and nitrogen sources. Our single Cloacimonadota MAG was present in low abundance at site U1545B in metagenomes between 25.8 and 63.8 mbsf. The Cloacimonadota MAG was not predicted to contain the capacity for carbon fixation, however our data showed (and the MetaPathPredict successfully predicted) that this taxon encodes genes for fermentation via acetate production (*pta*, *ack*), and synthesis of vitamin B7 and salvage of thiamine (vitamin B1), an indispensable cofactor in amino acid and carbohydrate metabolism. Further, salvage of B1 is linked to the metabolism of pyrimidines essential for maintenance and synthesis of the DNA strands (Gonçalves and Gonçalves, 2019). Biosynthesis of purines and pyrimidines is a core metabolic process, and was detected, and predicted, in our Cloacimonadota MAG.

Accounting for Seawater and Laboratory Contamination

Deep-sea drilling employs a mixture of seawater and lubricant mud during drilling operations, which carries seawater-derived microbial contaminants into the sediment samples. Contamination monitoring (using polyfluorinated chemical tracers) was run throughout the drilling operations on selected samples (Lever et al. 2006), yet not every sediment sample of the thousands that are collected during an IODP expedition can be tested. It is therefore necessary to account for the potential presence of mixed microbial communities derived from seawater and drilling lubricant by sequencing a "drilling fluid control" and excluding the detected sequences from our metagenomic data. The second type of contaminants are introduced through DNA extraction kits, reagents and handling (the "kitome", e.g., Salter et al., 2014). This contaminant community needs to be accounted for based on blank extractions ("kit/method control") where no sediment sample is added. Because DNA for metagenomes was extracted in two different labs, the Amend lab (USC) and the Edgcomb lab (WHOI), our study includes two blank extraction controls. Contaminants from all three controls were identified by mapping control reads to the metagenome assembly and removing all contigs that received mapping with minimum 98% identity over minimum 75% of the read length. Contamination control samples are listed in Supplementary Dataset 2, and contaminant MAGs in Supplementary Dataset 3.

Supplementary Figures

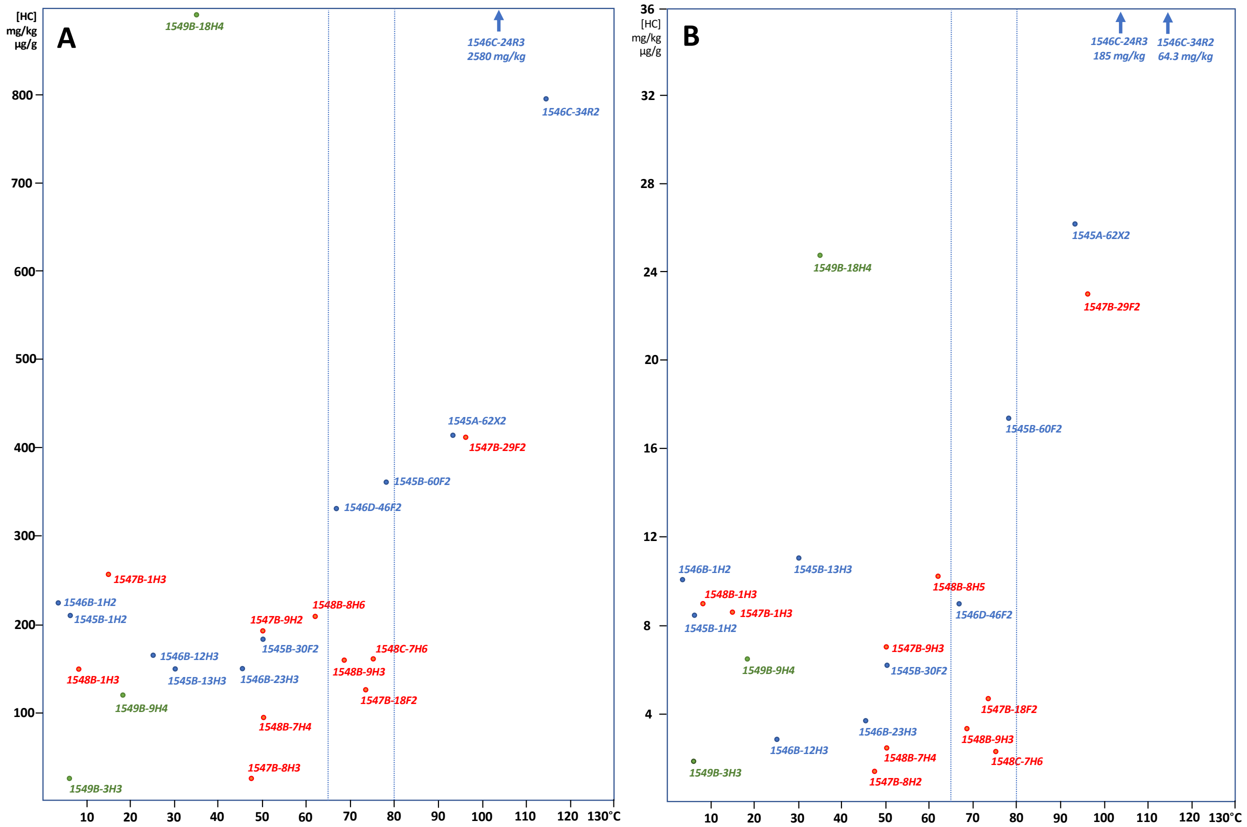

Supplementary Figure 1. **Hydrocarbon content of Guaymas Basin sediments**. A) Total petroleum hydrocarbon (C9-C44) content. B) Total saturated hydrocarbon content. Samples were analyzed at Alpha Analytical (Mansfield, MA, USA) for fingerprinting diagnostic compounds using EPA method 8015 (GC-FID; saturates) and a modified method 8270D (GCMS; PAHs), as detailed in Stout 2016. Hydrocarbon concentrations used in this figure are provided in the Source Data file.

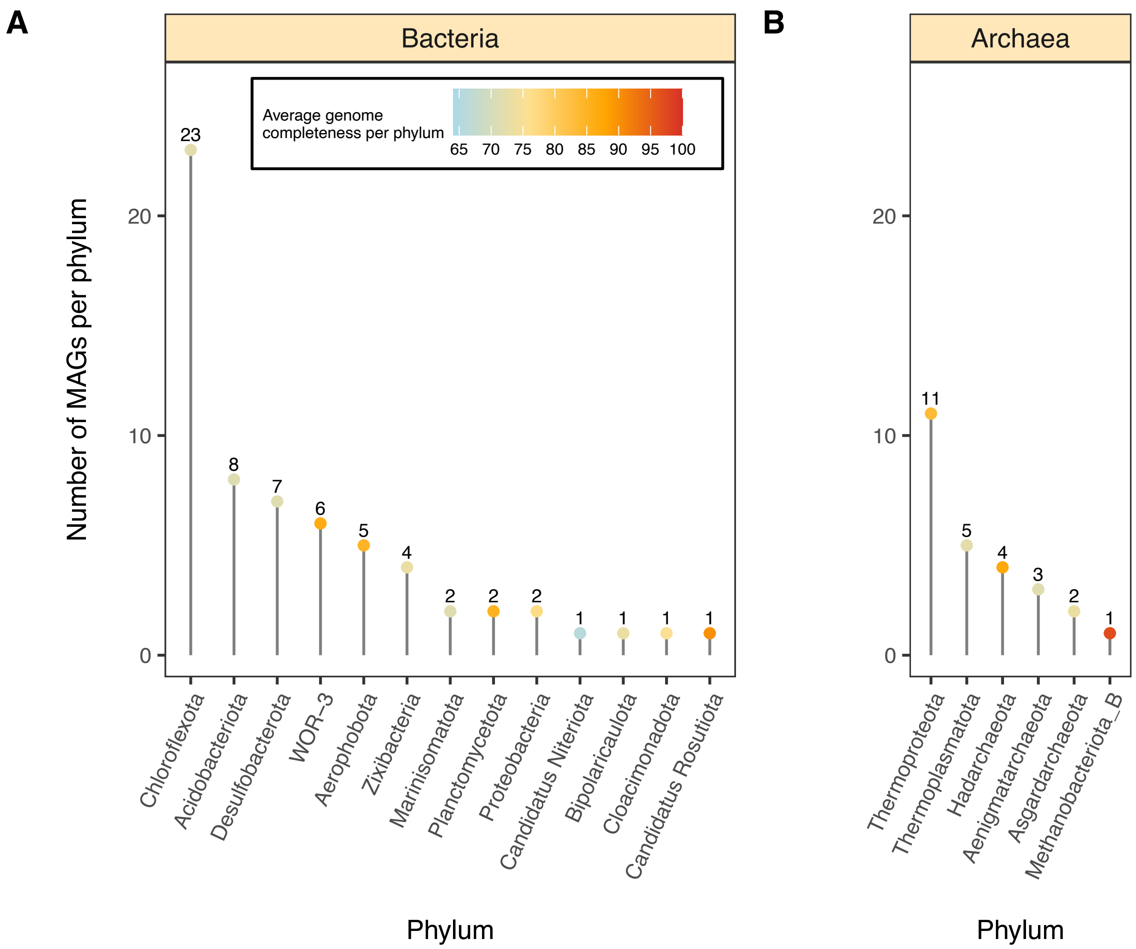

Supplementary Figure 2. **MAG recovery frequency**. Frequency of Guaymas Basin prokaryotic MAGs (≥ 50% completeness, ≤ 10% contamination) by bacterial (**A**) and archaeal phylum (**B**). Colored dots at the end of each line segment correspond to the mean genome completeness of the phylum; the number above the dot quantifies the number of genomes recovered from the phylum.

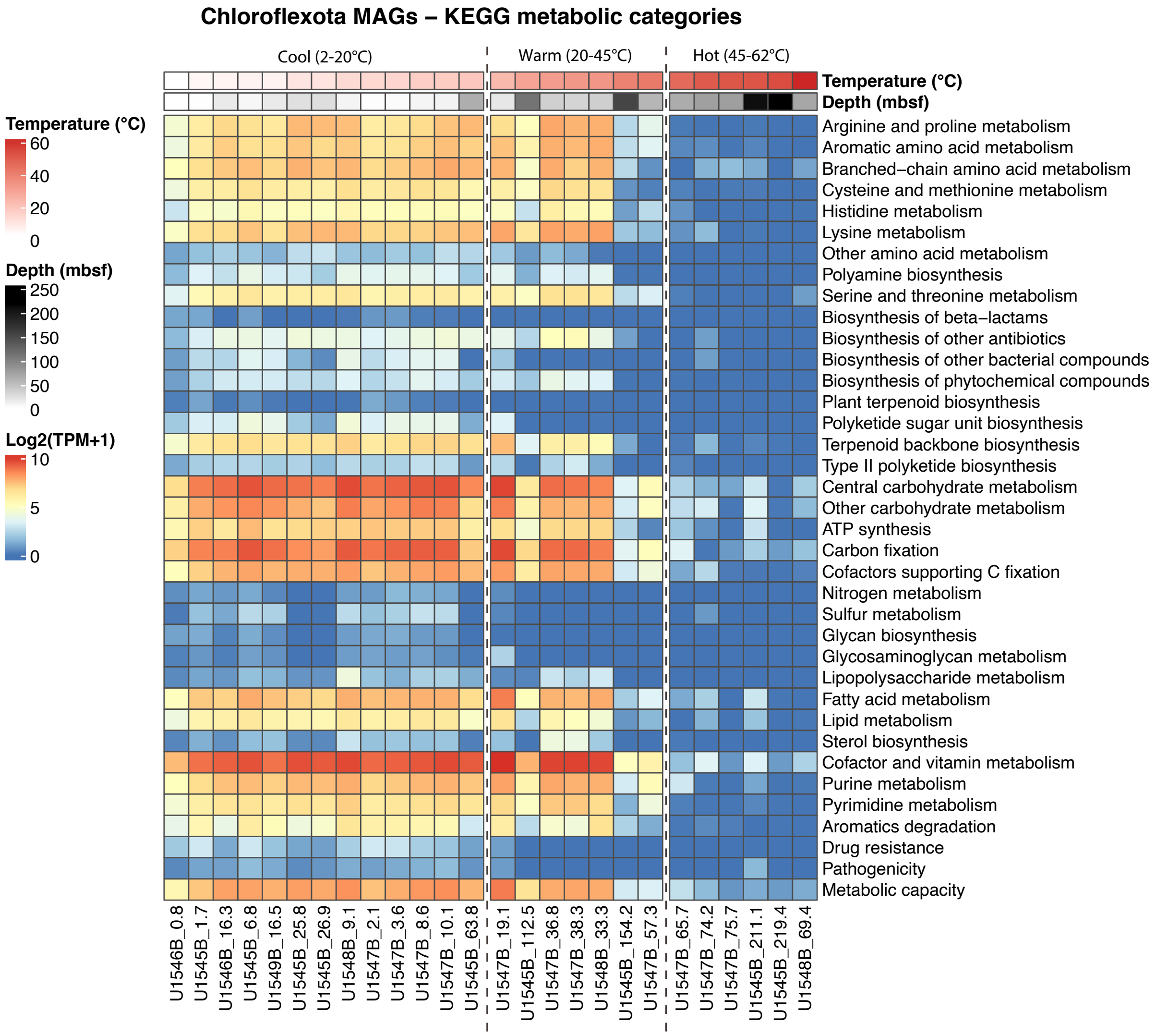

Supplementary Figure 3. Heatmap of read frequency for metabolic and cellular processes of Chloroflexi in Guaymas Basin metagenome samples. Metabolic and cellular processes were identified at the examined sites/depths using KofamScan. Processes involve pathways associated with energy-related metabolisms, genetic maintenance, survival strategies, and co-factor/vitamin biosynthesis. Sites and depths (mbsf) are given along the Y axis of the heatmap. Expression levels are normalized as log2 transformation of TPM+1 (a value of 1 was added to TPM values to avoid zeros). TPM: Transcripts Per Million.

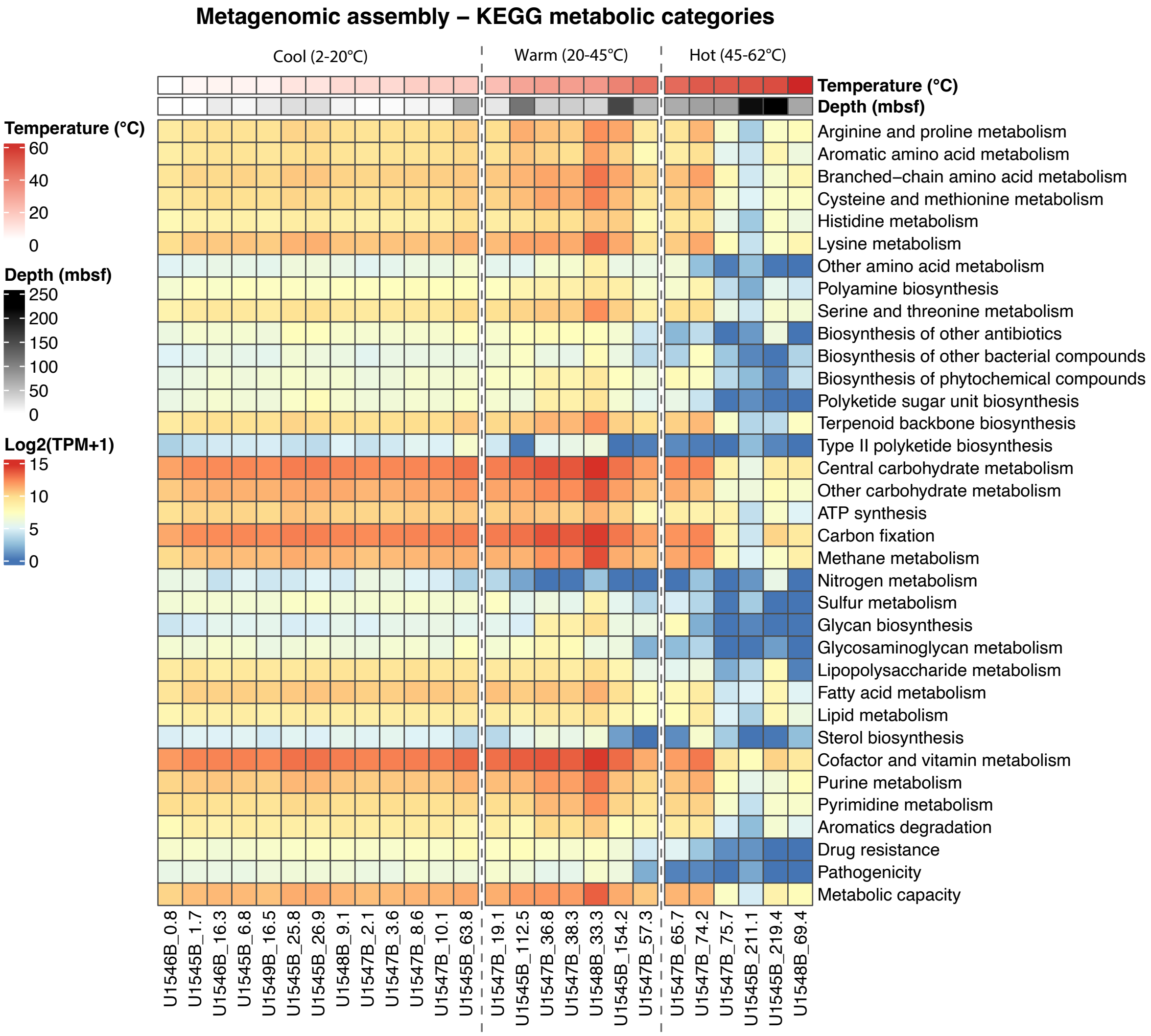

Supplementary Figure 4. Heatmap of read frequency for metabolic and cellular processes of the subsurface microbial community in Guaymas Basin metagenome samples. Metabolic and cellular processes were identified at the examined sites/depths using KofamScan. Processes involve pathways associated with energy-related metabolisms, genetic maintenance, survival strategies, and co-factor/vitamin biosynthesis. Sites and depths (mbsf) are given along the Y axis of the heatmap. Expression levels are normalized as log2 transformation of TPM+1 (a value of 1 was added to TPM values to avoid zeros). TPM, Transcripts Per Million.

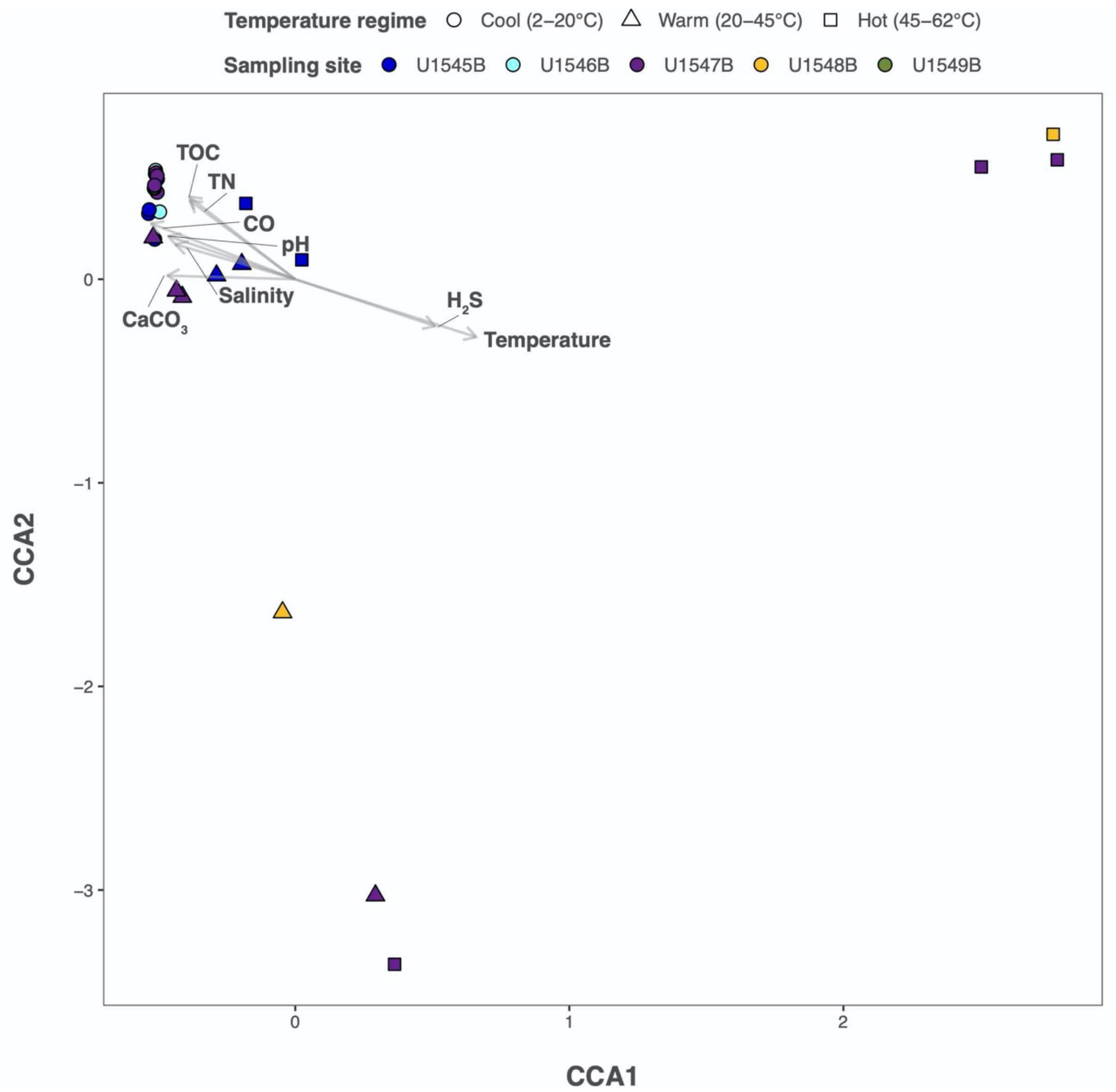

Supplementary Figure 5. Canonical Correlation Analysis (CCA) of subsurface MAGs and environmental parameters. The CCA plot depicts the correlation of Guaymas Basin MAG occurrence with in-situ environmental parameters. On the basis of Fisher's method for combining p-values, we show environmental variables with p-values < 0.05 resulting from a two-sided permutation test. Arrow direction indicates a positive or negative correlation of the environmental parameter with the ordination axes, for statistically significant ($p < 0.05$) environmental parameters (temperature, $p = 0.0001$; pH, $p = 0.0102$; salinity, $p = 0.0268$; hydrogen sulfide (H_2S), $p = 0.0053$; carbon monoxide (CO), $p = 0.004$; calcium carbonate ($CaCO_3$), $p = 0.0240$; total organic carbon

(TOC), p = 0.0084; total nitrogen (TN), p = 0.0103). Arrow length reflects correlation strength between environmental parameter and MAG occurrence. Samples are color-coded by site, and their temperature regimes are indicated by shape (circles for 2-20°C; triangles for 20-45°C and squares for 45-62°C).

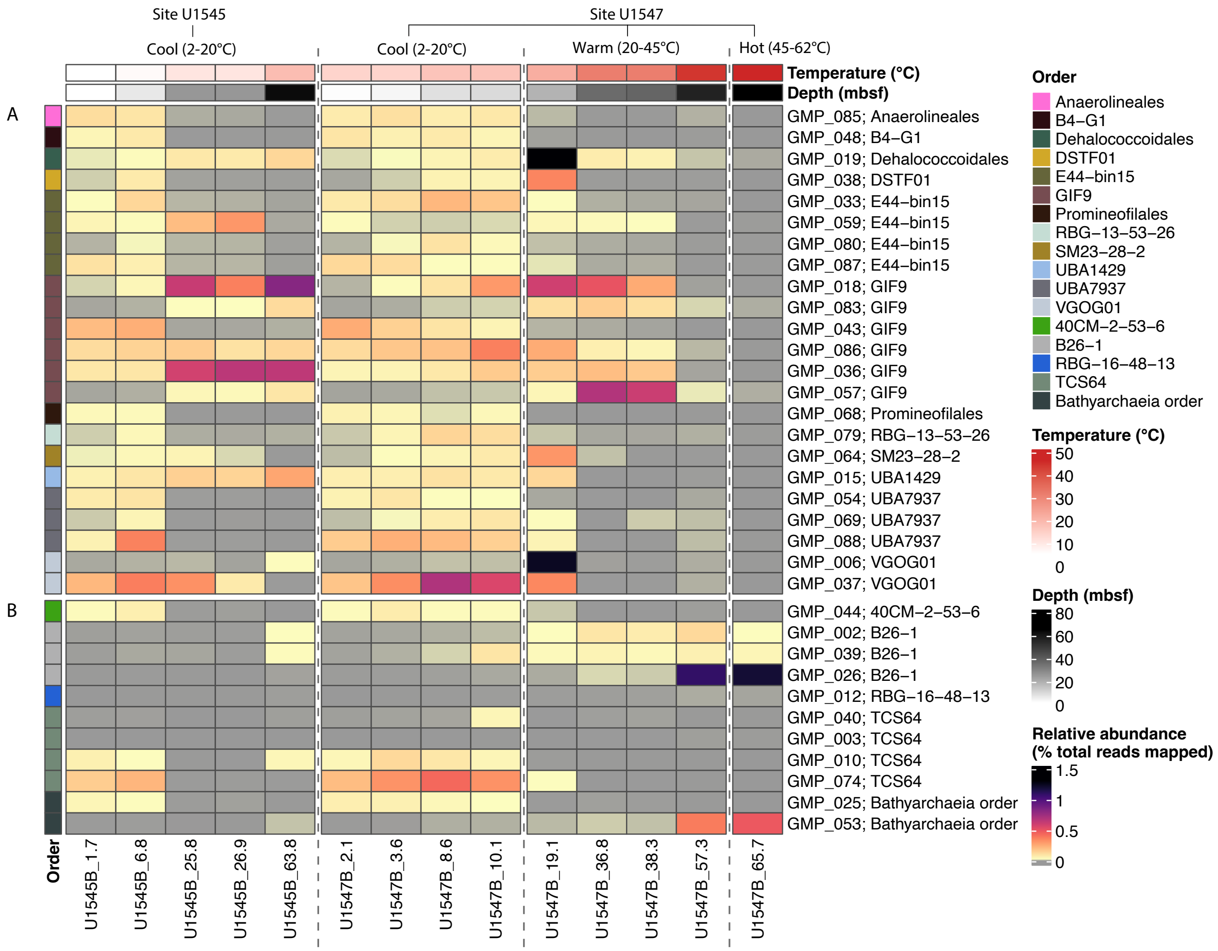

Supplementary Figure 6. Relative abundance of Chloroflexota and Thermoproteota MAGs, identified by order, in metagenomic samples from drill sites U1545B and U1547B. The heatmap for Chloroflexota (**A**) and Thermoproteota (**B**) shows for each column the percentage of total pre-processed reads from a metagenomic sample that mapped to all Chloroflexota and Thermoproteota MAGs, respectively, in order of increasing sampling depth and temperature from

left to right for samples from sites U1545B (left) and U1547B (right). MAG taxonomy is color coded to the left of the plot; taxonomic order names are given in the legend to the right of the heatmap. Temperatures and depths are color coded at the top of the plot.

Supplementary References

Altshuler, I., Raymond-Bouchard, I., Magnuson, E., Tremblay, J., Greer, C.W., Whyte, L.G. (2022). Unique high Arctic methane metabolizing community revealed through in situ 13CH4-DNA-SIP enrichment in concert with genome binning. *Scientific Reports*, *12*, 1160.

Anantharaman, K., Hausmann, B., Jungbluth, S.P., Kantor, R.S., Lavy, A., Warren, L. A., Rappé, M.S., Pester, M., Loy, A., Thomas, B.C. (2018). Expanded diversity of microbial groups that shape the dissimilatory sulfur cycle. *The ISME Journal*, *12*, 1715–1728.

Baker, B.J., Lazar, C.S., Teske, A.P., Dick, G.J. (2015). Genomic resolution of linkages in carbon, nitrogen, and sulfur cycling among widespread estuary sediment bacteria. *Microbiome*, *3*, 1–12.

Bonato, P., Alves, L.R., Osaki, J.H., Rigo, L.U., Pedrosa, F.O., Souza, E.M., Zhang, N., Schumacher, J., Buck, M., Wassem, R. (2016). The NtrY–NtrX two-component system is involved in controlling nitrate assimilation in *Herbaspirillum seropedicae* strain SmR1. *The FEBS Journal*, *283*, 3919–3930.

Buessecker, S., Palmer, M., Lai, D., Dimapilis, J., Mayali, X., Mosier, D., Jiao, J.-Y., Colman, D.R., Keller, L. M., St. John, E. (2022). An essential role for tungsten in the ecology and evolution of a previously uncultivated lineage of anaerobic, thermophilic Archaea. *Nature Communications*, *13*, 3773.

Castelle, C.J., Hug, L.A., Wrighton, K.C., Thomas, B.C., Williams, K.H., Wu, D., Tringe, S.G., Singer, S.W., Eisen, J.A., Banfield, J.F. (2013). Extraordinary phylogenetic diversity and metabolic versatility in aquifer sediment. *Nature Communications*, *4*, 2120.

Chatterjee, M., Fan, Y., Cao, F., Jones, A. A., Pilloni, G., Zhang, X. (2021). Proteomic study of *Desulfovibrio ferrophilus* IS5 reveals overexpressed extracellular multi-heme cytochrome associated with severe microbiologically influenced corrosion. *Scientific Reports*, *11*, 1–11.

Chklovski, A., Parks, D.H., Woodcroft, B.J., Tyson, G.W. (2022). CheckM2: a rapid, scalable and accurate tool for assessing microbial genome quality using machine learning. *BioRxiv*, 2022–2027.

Colognori, D., Trinidad, M., Doudna, J.A. (2023). Precise transcript targeting by CRISPR-Csm complexes. *Nat Biotechnol*. 41, 1256-1264.

DelVecchio, V.G., Kapatral, V., Elzer, P., Patra, G., Mujer, C. (2002). The genome of *Brucella melitensis*. *Veterinary Microbiology*, *90*, 587–592.

Deng, X., Okamoto, A. (2018). Electrode potential dependency of single-cell activity identifies the energetics of slow microbial electron uptake process. *Frontiers in Microbiology*, *9*, 2744.

Deng, X., Dohmae, N., Nealson, K.H., Hashimoto, K., Okamoto, A. (2018). Multi-heme cytochromes provide a pathway for survival in energy-limited environments. *Science Advances*, *4*, eaao5682.

Dodsworth, J. A., Gevorkian, J., Despujos, F., Cole, J.K., Murugapiran, S. K., Ming, H., Li, W.-J., Zhang, G., Dohnalkova, A., Hedlund, B.P. (2014). *Thermoflexus hugenholtzii* gen. nov., sp. nov., a thermophilic, microaerophilic, filamentous bacterium representing a novel class in the Chloroflexi, Thermoflexia classis nov., and description of Thermoflexaceae fam. Nov. and Thermoflexales ord. nov. *International Journal of Systematic and Evolutionary Microbiology*, *64*, 2119–2127.

Dombrowski, N., Seitz, K.W., Teske, A.P., Baker, B.J. (2017). Genomic insights into potential interdependencies in microbial hydrocarbon and nutrient cycling in hydrothermal sediments. *Microbiome*, *5*, 1–13.

Dombrowski, N., Teske, A.P., Baker, B.J. (2018). Expansive microbial metabolic versatility and biodiversity in dynamic Guaymas Basin hydrothermal sediments. *Nature Communications*, *9*(1), 4999.

Dong, X., Greening, C., Rattray, J. E., Chakraborty, A., Chuvochina, M., Mayumi, D., Dolfing, J., Li, C., Brooks, J.M., Bernard, B.B. (2019). Metabolic potential of uncultured bacteria and archaea associated with petroleum seepage in deep-sea sediments. *Nature Communications*, *10*(1), 1816.

Edgcomb, V.P., Teske, A.P., Mara, P. (2022). Microbial hydrocarbon degradation in Guaymas Basin—exploring the roles and potential interactions of fungi and sulfate-reducing bacteria. *Frontiers in Microbiology* 13:831828, doi 10.3389/fmicb.2022.831828

Eisenhofer, R., Odriozola, I., Alberdi, A. Impact of microbial genome completeness on metagenomic functional inference. *ISME COMMUN.* 3: 12 (2023). https://doi.org/10.1038/s43705-023-00221-z

Evans, P.N., Boyd, J.A., Leu, A.O., Woodcroft, B.J., Parks, D.H., Hugenholtz, P., Tyson, G.W. (2019). An evolving view of methane metabolism in the Archaea. *Nature Reviews Microbiology*, *17*(4), 219–232.

Evans, P.N., Parks, D.H., Chadwick, G.L., Robbins, S.J., Orphan, V.J., Golding, S.D., Tyson, G.W. (2015). Methane metabolism in the archaeal phylum Bathyarchaeota revealed by genome-centric metagenomics. *Science*, *350*(6259), 434–438.

Farag, I.F., Biddle, J.F., Zhao, R., Martino, A.J., House, C.H., León-Zayas, R.I. (2020). Metabolic potentials of archaeal lineages resolved from metagenomes of deep Costa Rica sediments. *The ISME Journal*, *14*, 1345–1358.

Fincker, M., Huber, L.A., Orphan, V.J., Rappé, M.S., Teske, A., Spormann, A.M. (2020). Metabolic strategies of marine subseafloor Chloroflexi inferred from genome reconstructions. *Environmental Microbiology* 22, 3188-3203.

Flieder, M., Buongiorno, J., Herbold, C.W., Hausmann, B., Rattei, T., Lloyd, K.G., Loy, A., Wasmund, K. (2021). Novel taxa of Acidobacteriota implicated in seafloor sulfur cycling. *The ISME Journal*, *15*(11), 3159–3180.

Fullerton, H., Moyer, C.L. (2016). Comparative single-cell genomics of Chloroflexi from the Okinawa Trough deep-subsurface biosphere. *Applied and Environmental Microbiology*, *82*, 3000–3008.

Garber, A.I., Nealson, K.H., Okamoto, A., McAllister, S. M., Chan, C.S., Barco, R.A., Merino, N. (2020). FeGenie: a comprehensive tool for the identification of iron genes and iron gene neighborhoods in genome and metagenome assemblies. *Frontiers in Microbiology* 11:37.

Geller-McGrath, D., Konwar, K., Edgcomb, V.P., Pachiadaki, M., Roddy, J., Wheeler, T., McDermott, J.E. (2022). MetaPathPredict: A machine learning-based tool for predicting metabolic modules in incomplete bacterial genomes. *BioRxiv*, 2012–2022.

He, Y., Li, M., Perumal, V., Feng, X., Fang, J., Xie, J., Sievert, S.M., Wang, F. (2016). Genomic and enzymatic evidence for acetogenesis among multiple lineages of the archaeal phylum Bathyarchaeota widespread in marine sediments. *Nature Microbiology*, *1*(6), 1–9.

Hernández, V.M., Arteaga, A., Dunn, M.F. (2021). Diversity, properties and functions of bacterial arginases. *FEMS Microbiology Reviews*, *45*, fuab034.

Islam, Z.F., Cordero, P.R.F., Feng, J., Chen, Y.-J., Bay, S.K., Jirapanjawat, T., Gleadow, R.M., Carere, C.R., Stott, M.B., Chiri, E. (2019). Two Chloroflexi classes independently evolved the ability to persist on atmospheric hydrogen and carbon monoxide. *The ISME Journal*, *13*, 1801–1813.

Jiang, Y., Shi, M., Shi, L. (2019). Molecular underpinnings for microbial extracellular electron transfer during biogeochemical cycling of earth elements. *Science China Life Sciences*, *62*, 1275–1286.

Jeuken, L. J. C., Hards, K., Nakatani, Y. (2020). Extracellular electron transfer: respiratory or nutrient homeostasis? *Journal of Bacteriology*, *202*, e00029-20.

Kapili, B.J., Barnett, S.E., Buckley, D.H., Dekas, A.E. (2020). Evidence for phylogenetically and catabolically diverse active diazotrophs in deep-sea sediment. *ISME J* 14, 971–983.

King, G. M. (2006). Nitrate-dependent anaerobic carbon monoxide oxidation by aerobic CO-oxidizing bacteria. *FEMS Microbiology Ecology*, *56*, 1–7.

Kishida, K., Sohrin, Y., Okamura, K., Ishibashi, J. (2004). Tungsten enriched in submarine hydrothermal fluids. *Earth and Planetary Science Letters*, *222*, 819–827

Kletzin, A., Adams, M.W.W. (1996). Tungsten in biological systems. *FEMS Microbiology Reviews*, *18*, 5–63.

Knittel, K., Boetius, A. (2009). Anaerobic oxidation of methane: progress with an unknown process. *Annual Review of Microbiology*, *63*, 311–334.

Kochetkova, T.V, Rusanov, I.I., Pimenov, N.V, Kolganova, T.V, Lebedinsky, A.V, Bonch-Osmolovskaya, E.A., Sokolova, T.G. (2011). Anaerobic transformation of carbon monoxide by microbial communities of Kamchatka hot springs. *Extremophiles*, *15*, 319–325.

Kung, J. W., Löffler, C., Dörner, K., Heintz, D., Gallien, S., van Dorsselaer, A., Friedrich, T., Boll, M. (2009). Identification and characterization of the tungsten-containing class of benzoyl-coenzyme A reductases. *Proceedings of the National Academy of Sciences*, *106*(42), 17687–17692.

Lever, M.A., Alperin, M., Inagaki, F., Nakagawa, S., Steinsbu, B.O., Teske, A., and IODP Expedition 301 Scientists. (2006). Trends in basalt and sediment core contamination during IODP Expedition 301. *Geomicrobiology Journal* 23, 517-530.

Light, S.H., Su, L., Rivera-Lugo, R., Cornejo, J. A., Louie, A., Iavarone, A.T., Ajo-Franklin, C. M., Portnoy, D. A. (2018). A flavin-based extracellular electron transfer mechanism in diverse Gram-positive bacteria. *Nature*, *562*, 140–144.

Lin,Y.-S., Heuer, V., Goldhammer, T., Kellermann, M.Y., Zabel, M., Hinrichs, K.U. (2012a). Towards constraining H_2 concentration in subseafloor sediment: a proposal for combined analysis by two distinct approaches. *Geochim. Cosmochim. Acta* 77, 186–201.

Lin, X., Kennedy, D., Fredrickson, J., Bjornstad, B., Konopka, A. (2012b). Vertical stratification of subsurface microbial community composition across geological formations at the Hanford Site. *Environmental Microbiology*, *14*, 414–425.

Liu, R., Wei, X., Song, W., Wang, L., Cao, J., Wu, J., Thomas, T., Jin, T., Wang, Z., Wei, W. (2022a). Novel Chloroflexi genomes from the deepest ocean reveal metabolic strategies for the adaptation to deep-sea habitats. *Microbiome*, *10*(1), 1–17.

Liu, S., Yu, S., Lu, X., Yang, H., Li, Y., Xu, X., Lu, H., Fang, Y. (2022b). Microbial communities associated with thermogenic gas hydrate-bearing marine sediments in Qiongdongnan Basin, South China Sea. *Frontiers in Microbiology*, 13:1032851.

Magnabosco, C., Ryan, K., Lau, M., Kuloyo, O., Lollar, S., Kieft, T., van Heerden E., Onstott, T.C. (2016). A metagenomic window into carbon metabolism at 3 km depth in Precambrian continental crust. *ISME J* 10, 730–741.

McGonigle, J. M., Lang, S. Q., Brazelton, W. J. (2020). Genomic evidence for formate metabolism by Chloroflexi as the key to unlocking deep carbon in Lost City microbial ecosystems. *Applied and Environmental Microbiology*, *86*, e02583-19.

Momper, L., Jungbluth, S. P., Lee, M. D., Amend, J. P. (2017). Energy and carbon metabolisms in a deep terrestrial subsurface fluid microbial community. *The ISME Journal*, *11*, 2319–2333.

Murphy, C. L., Biggerstaff, J., Eichhorn, A., Ewing, E., Shahan, R., Soriano, D., Stewart, S., VanMol, K., Walker, R., Walters, P. (2021). Genomic characterization of three novel Desulfobacterota classes expand the metabolic and phylogenetic diversity of the phylum. *Environmental Microbiology*, *23*, 4326–4343.

Palmer, M., Covington, J.K., Zhou, E.-M., Thomas, S.C., Habib, N., Seymour, C.O., Lai, D., Johnston, J., Hashimi, A., Jiao, J.-Y. (2023). Thermophilic Dehalococcoidia with unusual traits shed light on an unexpected past. *The ISME Journal* 17, 952-966.

Pawlowski, K., Klosse, U., De Bruijn, F.J. (1991). Characterization of a novel *Azorhizobium caulinodans* ORS571 two-component regulatory system, NtrY/NtrX, involved in nitrogen fixation and metabolism. *Molecular and General Genetics MGG*, *231*, 124–138.

Oelgeschläger, E., Rother, M. (2008). Carbon monoxide-dependent energy metabolism in anaerobic bacteria and archaea. *Archives of Microbiology*, *190*, 257–269.

Orsi, W.D., Edgcomb, V.P., Christman, G.D., Biddle, J. F. (2013). Gene expression in the deep biosphere. *Nature*, *499*, 205–208.
Qi, Y.-L., Evans, P. N., Li, Y.-X., Rao, Y.-Z., Qu, Y.-N., Tan, S., Jiao, J.-Y., Chen, Y.-T., Hedlund, B. P., Shu, W.-S. (2021). Comparative genomics reveals thermal adaptation and a high metabolic diversity in "Candidatus Bathyarchaeia. *Msystems*, *6*(4), e00252-21.
Salter, S.J., Cox, M.J., Turek, E.M., Calus, S.T., Cook, W.O., Moffatt, M.F., Turner, P., Parkhill, J., Loman, N.J., Walker, A.W. (2014). Reagent and laboratory contamination can critically impact sequence-based microbiome analyses. *BMC Biol* **12**, 87.
Saunders, J.K., Fuchsman, C.A., McKay, C., Rocap, G. (2019). Complete arsenic-based respiratory cycle in the marine microbial communities of pelagic oxygen-deficient zones. *Proceedings of the National Academy of Sciences*, *116*, 9925–9930.
Sekowska, A., Dénervaud, V., Ashida, H., Michoud, K., Haas, D., Yokota, A., Danchin, A. (2004). Bacterial variations on the methionine salvage pathway. *BMC Microbiology*, *4*, 1–17.
Shi, L., Dong, H., Reguera, G., Beyenal, H., Lu, A., Liu, J., Yu, H.-Q., Fredrickson, J.K. (2016). Extracellular electron transfer mechanisms between microorganisms and minerals. *Nature Reviews Microbiology*, *14*, 651–662.
Sieber, C.M.K., Probst, A.J., Sharrar, A., Thomas, B. C., Hess, M., Tringe, S.G., Banfield, J.F. (2018). Recovery of genomes from metagenomes via a dereplication, aggregation and scoring strategy. *Nature Microbiology*, *3*, 836–843.
Sirajuddin, S., Rosenzweig, A.C. (2015). Enzymatic oxidation of methane. *Biochemistry*, *54*, 2283–2294.
Sokolova, T.G., Henstra, A.-M., Sipma, J., Parshina, S.N., Stams, A.J.M., Lebedinsky, A.V. (2009). Diversity and ecophysiological features of thermophilic carboxydotrophic anaerobes. *FEMS Microbiology Ecology*, *68*, 131–141.
Speirs, L.B.M., Rice, D.T.F., Petrovski, S., Seviour, R.J. (2019). The phylogeny, biodiversity, and ecology of the Chloroflexi in activated sludge. *Frontiers in Microbiology*, *10*, 2015.
Speth, D.R., Yu, F.B., Connon, S.A., Lim, S., Magyar, J.S., Peña-Salinas, M.E., Quake, S.R., Orphan, V.J. (2022). Microbial communities of Auka hydrothermal sediments shed light on vent biogeography and the evolutionary history of thermophily. *The ISME Journal*, *16*, 1750–1764.
Stout, S. A. (2016). Oil spill fingerprinting method for oily matrices used in the Deepwater Horizon NRDA. *Environ. Forensics* 17, 218–243.
Suominen, S., Dombrowski, N., Sinninghe Damsté, J.S., Villanueva, L. (2021). A diverse uncultivated microbial community is responsible for organic matter degradation in the Black Sea sulphidic zone. *Environmental Microbiology*, *23*, 2709–2728.
Timmers, P.H.A., Welte, C.U., Koehorst, J.J., Plugge, C.M., Jetten, M.S.M., & Stams, A.J.M. (2017). Reverse methanogenesis and respiration in methanotrophic archaea. *Archaea*, *2017*: 1654237

Von Damm, K.L., Edmond, J.M., Measures, C.I., Grant, B. (1985). Chemistry of submarine hydrothermal solutions at Guaymas Basin, Gulf of California. *Geochimica et Cosmochimica Acta*, *49*, 2221–2237

Vanwonterghem, I., Evans, P.N., Parks, D.H., Jensen, P.D., Woodcroft, B.J., Hugenholtz, P., Tyson, G.W. (2016). Methylotrophic methanogenesis discovered in the archaeal phylum Verstraetearchaeota. *Nature Microbiology*, *1*, 1–9.

Venceslau, S.S., Stockdreher, Y., Dahl, C., Pereira, I.A.C. (2014). The "bacterial heterodisulfide" DsrC is a key protein in dissimilatory sulfur metabolism. *Biochimica et Biophysica Acta-Bioenergetics*, *1837*, 1148–1164.

Villanueva, L., von Meijenfeldt, F.A.B., Westbye, A.B., Yadav, S., Hopmans, E.C., Dutilh, B. E., Damsté, J.S.S. (2021). Bridging the membrane lipid divide: bacteria of the FCB group superphylum have the potential to synthesize archaeal ether lipids. *The ISME Journal*, *15*, 168–182.

Vuillemin, A., Kerrigan, Z., D'Hondt, S., Orsi, W.D. (2020). Exploring the abundance, metabolic potential and gene expression of subseafloor Chloroflexi in million-year-old oxic and anoxic abyssal clay. *FEMS Microbiology Ecology*, *96*, fiaa223.

Vorholt, J.A., Marx, C.J., Lidstrom, M.E., Thauer, R.K. (2000). Novel formaldehyde-activating enzyme in *Methylobacterium extorquens* AM1 required for growth on methanol. *Journal of Bacteriology*, *182*, 6645–6650.

Wegener, G., Laso-Pérez, R., Orphan, V. J., Boetius, A. (2022). Anaerobic degradation of alkanes by marine archaea. *Annual Review of Microbiology*, *76*, 553–577.

Wong, H.L., MacLeod, F.I., White, R.A., Visscher, P.T., Burns, B.P. (2020). Microbial dark matter filling the niche in hypersaline microbial mats. *Microbiome*, *8*, 1–14.

Xiong, L., Jian, H., Zhang, Y., Xiao, X. (2016). The two sets of DMSO respiratory systems of *Shewanella piezotolerans* WP3 are involved in deep sea environmental adaptation. *Frontiers in Microbiology*, *7*, 1418.

Zhang, W., Ding, W., Yang, B., Tian, R., Gu, S., Luo, H., Qian, P.-Y. (2016). Genomic and transcriptomic evidence for carbohydrate consumption among microorganisms in a cold seep brine pool. *Frontiers in Microbiology*, *7*, 1825.

Zhou, Z., St John, E., Anantharaman, K., Reysenbach, A.-L. (2022). Global patterns of diversity and metabolism of microbial communities in deep-sea hydrothermal vent deposits. *Microbiome*, *10*, 1–22.
