# Supplementary material for: Metagenomic profiles of archaea and bacteria within thermal and geochemical gradients of the Guaymas Basin deep subsurface
Source: Nat Commun. 2023 Nov 27;14:7768. doi: 10.1038/s41467-023-43296-x (PMC10681998; doi:10.1038/s41467-023-43296-x)
Supplement: Supplementary file 3 — Description of Additional Supplementary Files [file 41467_2023_43296_MOESM3_ESM.pdf]

Description of Additional Supplementary Files:

Supplementary Data 1: Geochemical data from sites and depths where metagenomic data are available. The columns from left to right include: sample name, Depth (in meters below seafloor; mbsf), Temperature (°C), pH, Alkalinity (mM), Salinity, SO_4^{2-} (mM), PO_4^{3-} (µM), H_2S (µM), NH_4^+ (mM), Mg^{2+} (mM), Ba^{2+} (µM), CH_4 (mM), H_2 (nM), CO (nM), DOC (mg/L), DIC (mM), $CaCO_3$ (wt%), TOC (wt%), TN (wt%), TOC/TN, Fe (µM), Total Petroleum Hydrocarbons (mg/kg sediment), Total saturated hydrocarbons (mg/kg sediment), total polyaromatic hydrocarbons (µg/kg sediment). Temperature values were interpolated for each sample using linear regression of the local thermal gradient (°C/m) multiplied by depth (mbsf), plus the y-axis intercept: U1545B, T = 0.225 x depth + 4.899; U1546B, T = 0.221 x depth + 2.627; U1547B, T = 0.511 x depth + 13.01; U1548B, T = 0.804 x depth + 6.499; U1549A/B, T = 0.194 x depth + 3.532.

Supplementary Data 2: Metagenomic and metatranscriptomic sequencing data. Spreadsheet “MetagenomeSeqDepth” contains the metagenomic read counts of each sample before and after quality control (QC). The DNA concentration is expressed in nanograms of DNA per gram of sampled sediment. The sediment depth is given in meters below seafloor (mbsf). Kb, kilobase pairs. Spreadsheet “MetatranscriptomeSeqDepth” contains metatranscriptomic read counts before and after quality control (QC), the quality score "q20 rate" (% of bases whose accuracy exceeds 99%), and the total recovered RNA (ng μl^{-1}) from each sample. Depth is given in meter below sea floor (mbsf). ND indicates that RNA concentration was below the detection limit using Qubit RNA High Sensitivity (HS) assay kit.

Supplementary Data 3: Taxonomy and assembly statistics of removed putative contaminant MAGs. Column A indicates the names of the bins identified as “contaminants” in this study; Columns B-C show the estimated % of completeness and contamination of these MAGs using CheckM2 (Chklovski et al., 2022a). Columns D-J provide taxonomic classification for the MAGs. Column K-M include metrics for quality score ("das_tool_bin_score"), completeness (based on presence/absence of single copy genes; "das_tool_scg_completeness"), and the redundancy (expressed as single copy gene duplicates; "das_tool_scg_redundancy") in each MAG (Sieber et al., 2018). Columns N-P provide the genome size, the number of contigs and the N50 score of each MAG.

Supplementary Data 4: Taxonomy and assembly statistics of all 89 metagenomeassembled genomes (MAGs). Columns A-C indicate the names of the 89 MAGs used in this study, their estimated completeness and contamination using CheckM2 (Chklovski et al., 2022a). Columns D-J provide taxonomic classifications for the MAGs. Column K-M include metrics on the quality score ("das_tool_bin_score"), the completeness (based on presence/absence of single copy genes; "das_tool_scg_completeness"), and the redundancy (expressed as single copy gene duplicates; "das_tool_scg_redundancy") in each MAG (Sieber et al., 2018). Columns N-P provide the genome_size, the number of contigs and the N50 score of each MAG.

Supplementary Data 5: KofamScan gene annotations for all medium quality MAGs discussed in this study (≥ 50% completeness, ≤ 10% contamination: n = 89). Columns A-H contains the name of the Bacterial and Archaeal MAGs discussed in this study (n = 89) and their taxonomic classifications. Columns I-P indicate with an (*) those sequence matches with scores above the adaptive score threshold; the name of the predicted gene from the output of Prodigal gene calling on each of the MAGs; the KEGG Orthology k number for each gene annotation; the e-value score of the HMM alignment; the HMM alignment score; the suggested HMM alignment score threshold for a robust annotation; the full gene name information for the annotation ("KO definition"); if the predicted gene is identified based on the HMM models as full protein sequence" ("full") or a specific protein domain ("domain").

Supplementary Data 6: Spreadsheet "Supplementary Table 6a" contains the gene annotations provided by Prokka for the 89 MAGs discussed in this study (≥ 50% completeness, ≤ 10% contamination). Columns A-E contain the MAG names; the name of predicted gene from the output of Prodigal gene calling on each of the MAGs ("gene_name"); the start and end positions of each gene sequence; the DNA strand where the predicted gene was identified. Columns F-J indicate the abbreviated and the full gene name ("hypothetical protein" denotes an unknown protein); the database used to make the gene annotation ("dbxref"); the enzyme commission number ("ec_number"); the name of the contig ("contig") where the gene is located on. Columns K-Q provide taxonomic classifications of MAGs. Spreadsheet "Supplementary Table 6b" provides the functional annotations of the predicted genes for the 89 MAGs discussed in this study (≥ 50% completeness, ≤ 10% contamination). Columns A-C contain the name of the Bacterial and Archaeal MAGs and their taxonomic classifications in phylum and order level. Columns D-G provide the metabolic category associated with the annotated gene; the predicted gene function; the abbreviated and the full name of the predicted gene. Columns H-N denote the HMM files used to make the gene annotation; the KEGG ortholog identifier ("Corresponding.KO"); the reaction, substrate and product that the encoded gene is involved in; the threshold cutoffs for each hmm ("Hmm.detecting.threshold"); the gene that received a hit to the HMMs.

Supplementary Data 7: AntiSMASH 6.0 biosynthetic gene cluster annotations for the 89 MAGs discussed in this study (≥ 50% completeness, ≤ 10% contamination). Columns A-E contain the name of the Bacterial and Archaeal MAGs that contained BGCs > 5 kilobases in size; the taxonomic affiliation (domain, phylum, class, and order) of each of the MAGs. Columns F-I contain the BGC name; the start and end positions of the cluster; the DNA strand where the BGC was on. Column J-L contain the product and length of BGC; the contig where the BGC was detected.

Supplementary Data 8: Abundances of KEGG metabolic categories for all recovered Chloroflexota MAGs. Abundances are normalized as Transcript per million (TPM). Each abundance category corresponds to the total (normalized) read recruitment to genes of Chloroflexota MAGs within each KEGG module class across all metagenomic samples (in columns 2-27). Samples are named using the site and depth from where they were collected.

Supplementary Data 9: Abundances of KEGG metabolic categories for all metagenomic samples. Abundances are normalized as transcript per million (TPM). Each abundance category corresponds to the total (normalized) read recruitment to genes within each KEGG module class across all metagenomic samples. Samples are named using the site and depth from where they were collected.

Supplementary Data 10: MetaPathPredict output for Cloacimonadota and Zixibacteria bacterial MAGs. The output table depicts the predicted presence or absence of KEGG metabolic modules (n = 476) for one Cloacimonadota and 4 Zixibacteria bacterial MAGs. Columns A-C indicate the metabolic category where the KEGG module belongs (if any, otherwise it is labelled as "No module class"); the name of each KEGG module; the unique numeric identifier of each KEGG module used in the KEGG database. Columns D-H indicate presence/absence predictions for the one Cloacimonadota and the 4 Zixibacteria MAGs. The follow classifications are used in the colorcoded (red/green) cells of these columns: "present: predicted" (the module is present in the genome and was predicted present by MetaPathPredict), "present: not predicted" (the module is present in the genome and was predicted absent), "absent: not predicted" (the module is completely absent in the genome and was predicted absent), "absent: predicted" (the module is completely absent in the genome and was predicted present), "incomplete: not predicted" (the module is partially present in the genome and was predicted absent), and "incomplete: predicted" (the module is partially present in the genome and was predicted present).
