# Supplementary material for: Metagenomic profiles of archaea and bacteria within thermal and geochemical gradients of the Guaymas Basin deep subsurface
Source: Nat Commun. 2023 Nov 27;14:7768. doi: 10.1038/s41467-023-43296-x (PMC10681998; doi:10.1038/s41467-023-43296-x)
Supplement: Supplementary file 14 — Reporting Summary [file 41467_2023_43296_MOESM14_ESM.pdf]

nature portfolio

Corresponding author(s): Andreas Teske
Last updated by author(s): Oct 10, 2023

Reporting Summary

Nature Portfolio wishes to improve the reproducibility of the work that we publish. This form provides structure for consistency and transparency in reporting. For further information on Nature Portfolio policies, see our Editorial Policies and the Editorial Policy Checklist.

Statistics

For all statistical analyses, confirm that the following items are present in the figure legend, table legend, main text, or Methods section.

n/a	Confirmed	
☐	☒	The exact sample size (n) for each experimental group/condition, given as a discrete number and unit of measurement
☒	☐	A statement on whether measurements were taken from distinct samples or whether the same sample was measured repeatedly
☐	☒	The statistical test(s) used AND whether they are one- or two-sided<br>*Only common tests should be described solely by name; describe more complex techniques in the Methods section.*
☒	☐	A description of all covariates tested
☒	☐	A description of any assumptions or corrections, such as tests of normality and adjustment for multiple comparisons
☐	☒	A full description of the statistical parameters including central tendency (e.g. means) or other basic estimates (e.g. regression coefficient) AND variation (e.g. standard deviation) or associated estimates of uncertainty (e.g. confidence intervals)
☐	☒	For null hypothesis testing, the test statistic (e.g. F, t, r) with confidence intervals, effect sizes, degrees of freedom and P value noted<br>*Give P values as exact values whenever suitable.*
☒	☐	For Bayesian analysis, information on the choice of priors and Markov chain Monte Carlo settings
☒	☐	For hierarchical and complex designs, identification of the appropriate level for tests and full reporting of outcomes
☒	☐	Estimates of effect sizes (e.g. Cohen's d, Pearson's r), indicating how they were calculated

Our web collection on statistics for biologists contains articles on many of the points above.

Software and code

Policy information about availability of computer code

Data collection	No software was used.
Data analysis	Custom command line scripts, R scripts, and Python scripts used in data analysis for this project are available in the following GitHub repository: https://github.com/d-mcgrath/guaymas_basin (DOI: 10.5281/zenodo.8422630). The following open-source softwares were used: CoverM 0.6.1, GTDB-Tk 2.1.0, CheckM2 1.0.0, antiSMASH 6.0, BWA 2.0 aligner, Trimmomatic v0.39, MEGAHIT 1.2.9, MetaBAT2 2.12.183, MaxBin2 2.2.7, CONCOCT 1.1.0, DAS Tool 1.1.6, Prodigal 2.6.3, Prokka 1.14.6, KofamScan 1.3.0, METABOLIC 4.0 Samtools 1.16.1, Minimap2 2.24-r1122, ggplot2 3.3.6, tidyverse 1.3.2, FastQC v.0.11.9, vegan 2.6-4 package.

For manuscripts utilizing custom algorithms or software that are central to the research but not yet described in published literature, software must be made available to editors and reviewers. We strongly encourage code deposition in a community repository (e.g. GitHub). See the Nature Portfolio guidelines for submitting code & software for further information.

Data

Policy information about availability of data

All manuscripts must include a data availability statement. This statement should provide the following information, where applicable:
- Accession codes, unique identifiers, or web links for publicly available datasets
- A description of any restrictions on data availability
- For clinical datasets or third party data, please ensure that the statement adheres to our policy

The raw metagenome and metatranscriptome sequence data generated in this study have been deposited in the NCBI GenBank database under the Bioproject

accession number PRJNA909197 (https://www.ncbi.nlm.nih.gov/bioproject/PRJNA909197). Metatranscriptome reads are deposited under the accession numbers SRR22580929-SRR22580947. Metagenome reads are deposited under the accession numbers SRR22580794-SRR2258807 and SRR23614663-SRR236114677. Biogeochemical and thermal shipboard data for all IODP385 sites discussed in this study (U1545-U1552) are publicly available on the IODP Expedition 385 online report (http://publications.iodp.org/proceedings/385/385title.html). Shipboard data can be downloaded for each drilling site individually, as numbered excel tables. Post-cruise geochemical data sets (DIC, TOC, TN, hydrocarbons) have been submitted to the Biological and Chemical Oceanography database (BCO-DMO) and are publicly available under project number 833856 (https://www.bco-dmo.org/project/833856). Publicly available datasets used in this study include the CheckM2 database (https://zenodo.org/record/4626519), the GTDB-Tk database release R214 (https://ecogenomics.github.io/GTDBTk/installing/index.html), the KOfam database (ftp://ftp.genome.jp/pub/db/kofam/), the METABOLIC database (https://github.com/AnantharamanLab/METABOLIC), the MEROPS database (https://www.ebi.ac.uk/merops/download_list.shtml), the dbCAN2 database (http://bcb.unl.edu/dbCAN2/download/Databases/dbCAN-old@UGA/dbCAN-fam-HMMs.txt), ISfinder database (https://isfinder.biotoul.fr/), NCBI Bacterial Antimicrobial Resistance database (https://www.ncbi.nlm.nih.gov/bioproject/313047), UniProtKB (SwissProt) database (https://www.uniprot.org/uniprot/?query=reviewed:yes), Prokka databases (https://github.com/tseemann/prokka) and the antiSMASH 6.0 databases (https://dl.secondarymetabolites.org/releases/).

Research involving human participants, their data, or biological material

Policy information about studies with human participants or human data. See also policy information about sex, gender (identity/presentation), and sexual orientation and race, ethnicity and racism.

Reporting on sex and gender	NA
Reporting on race, ethnicity, or other socially relevant groupings	NA
Population characteristics	NA
Recruitment	NA
Ethics oversight	NA

Note that full information on the approval of the study protocol must also be provided in the manuscript.

Field-specific reporting

Please select the one below that is the best fit for your research. If you are not sure, read the appropriate sections before making your selection.

☐ Life sciences ☐ Behavioural & social sciences ☒ Ecological, evolutionary & environmental sciences

For a reference copy of the document with all sections, see nature.com/documents/nr-reporting-summary-flat.pdf

Ecological, evolutionary & environmental sciences study design

All studies must disclose on these points even when the disclosure is negative.

Study description	We used metagenomic and metatranscriptomic analyses, and cell counts to examine the distribution and activity of subsurface bacteria and archaea in the hydrothermally heated sediments of Guaymas Basin (Gulf of California, Mexico). We extracted/isolated DNA, total RNA and microbial cells from subsurface sediments collected during the IODP385 Expedition in Guaymas Basin (September 2019-November 2019). During this expedition, eight drilling sites with distinct temperature and geochemical profiles were drilled in an effort to study subsurface microbial communities hosted in Guaymas Basin and to expand our understanding of the conditions that limit life in the deep biosphere. The extracted DNA was submitted for metagenomic library prepartion at DNA Sequencing & Genotyping Center (University of Delaware). The constructed libraries were sequenced with NextSeq 550 (Illumina) at DNA Sequencing & Genotyping Center (University of Delaware), and with NovaSeq S4 PE150 (Illumina) at the Davis Genome Center (University of California). Our metagenomic analyses generated 89 medium quality metagenome-assembled genomes (MAGs; n = 89) with ≥ 50% completeness and ≤ 10% contamination that were kept for downstream analysis. The extracted total RNA was used for generating cDNA libraries (n=19) that were submitted to Georgia Genomics and Bioinformatics Core for sequencing using NextSeq 500 PE 150 High Output (Illumina). The metatranscriptome analyses created 640,136 assembled metatranscripts with size > 165 bp. Our study included "kit/method" controls that were processed as samples, and a "drilling fluid" control that was used as an additional control on our metagenomic analyses. Our study did not include biological and technical replicates. Non-metric multidimensional scaling (nMDS) ordination plot of MAGs and environmental parameters was chosen to depict correlation between MAG occurrence with the in-situ environmental parameters. Only those statistically significant ($p < 0.05$; plot stress: 0.106) environmental parameters (TOC, DIC, T, Salinity, Alkalinity etc) were fitted on the nMDS plot. The estimated genome size of the MAGs vs. a) temperature and b) sediment depth, was examined using two-sided partially overlapping samples t-tests comparing estimated genome sizes by temperature and depth regimes, and to estimate the adjusted p-values. We used the percentage of total pre-processed metatranscriptome reads (relative abundance) that mapped to all 89 MAGs to estimate the MAG metatranscriptomic read recruitment. We used the percentage of total pre-processed metagenomic reads (relative abundance) that mapped to all 89 MAGs to estimate the distribution of MAGs at three different temperature regimes (cold, warm, hot; 2-20°C, 20-45°C, 45-60°C).
Research sample	Sediment cores were collected during IODP Expedition 385 using the drilling vessel JOIDES Resolution during September to November 2019. Among the major aims of IODP385 was to study the subsurface microbial communities of Guaymas Basin, describe their metabolic activities in the deep biosphere and determine what controls their survival and distribution at those subsurface depths. For this reason, IODP 385 drilled eight sites with distinct biogeochemical and thermal profiles into the sedimented off-axis regions and axial trough of Guaymas Basin (sites U1545-U1552). Drilling holes at each site were first advanced using advanced piston

	coring (APC), then half-length APC, and then extended core barrel (XCB) coring as necessary. Overall, sites U1545 and U1546 are adjacent sites located ~52 km northwest of the northern Guaymas Basin axial trough. U1546 has a massive, thermally equilibrated sill between 350-430 mbsf that disrupts the sedimentary strata, and changes the physical properties and geochemical gradients of the sediment. Sites U1547 and U1548 are ~27 km northwest of the axial trough, where a shallow, recently emplaced hot sill creates steep thermal gradients and drives hydrothermal circulation. Off-axis sites U1549 and U1552 are methane cold seep sites that are driven by deeply buried old sill intrusions. Off-axis site U1550 is located within the northern axial trough, and site U1551 in the southeastern Guaymas Basin is most strongly influenced by terrigenous input from the Sonoran Margin. Sediments were collected for metagenomic and metatranscriptomic analyses, and cell counts from all 8 drilling sites and depths that extend from 0.8 meters below sea floor (mbsf) down to 219.4 mbsf, and with temperature regimes that span from 3.5°C to 62°C.
Sampling strategy	Overall, during IODP 385 eight sites with distinct geological and temperature profiles were drilled into the sedimented off-axis regions and axial trough of Guaymas Basin (sites U1545-U1552). Drilling holes at each site were first advanced using advanced piston coring (APC), then half-length APC, and then extended core barrel (XCB) coring as necessary. The sites were initially selected based on the Cruise EW0210 multichannel seismic profiles and the observations from Cruise AT15-54, and were adjusted based on results from the new seismic lines and two additional site survey cruises by the R/V El Puma (7–27 October 2014) and Atlantis (9–27 December 2016). The 2014 El Puma cruise (led by Chief Scientist Carlos Mortera, Universidad Nacional Autónoma de México [UNAM], Mexico) performed a detailed bathymetric survey of the central portion of the northern Guaymas Basin spreading segment and collected sediment piston cores 3–5 m in length from the northwestern side of the northern spreading segment, the Sonora margin, and the circular seep structure called Ringvent. These cores provided shallow subsurface sediments near several proposed Expedition 385 drill sites and enabled a preliminary geochemical and microbial characterization of the proposed off-axis drill sites. Numerous shallow sediment cores and seafloor grab samples were also collected during Sonne Cruise 241. A major discovery from that cruise was a large, active hydrothermal vent field located on the southeastern edge of the northern Guaymas Basin trough. The last site survey cruise on Atlantis studied the Ringvent sites (U1547 and U1548) with human occupied vehicle (HOV) Alvin Dives 4864 and 4865 and autonomous underwater vehicle (AUV) Sentry Dives 410 and 411 (led by Chief Scientist Andreas Teske, University of North Carolina at Chapel Hill, US) providing faunal observations, microbial analyses, thermal gradient measurements in surficial sediments, and mineralogic and pore water chemistry analyses that demonstrated this site is hydrothermally active. These results were used to further characterize the proposed drill sites and to adjust their locations. Most sediment cores collected in the IODP385 survey spanned the sulfate-reducing zone, the sulfate-methane transition zones (SMTZ), and the methanogenic zone from each site (deeper sampling depth at 211.1 mbsf). Temperature measurements were made along the retrieved sediment core using the advanced piston corer temperature (APCT-3) and Sediment Temperature 2 (SET2) tools, and 4 sediment subsamples of 50 ml (see section Data collection) were immediately collected and stored at -80°C, for metagenomic and metranscriptomic analyses. As mentioned the preliminary expeditions provided precious insights on site locations, geochemical data and microbial characterization that were necessary to organize the IODP385 sampling strategy, and the collected sediment volumes.
Data collection	Temperature measurements of the collected sediment cores was performed using the advanced piston corer temperature (APCT-3) and Sediment Temperature 2 (SET2) tools. Downhole logging conducted after coring used the triple combination and Formation MicroScanner sonic logging tool strings. After bringing core sections onto the core receiving platform of the D/V JOIDES Resolution, whole round samples for microbiology were retrieved within ~30 minutes using ethanol-cleaned spatulas. Samples for biogeochemical measurements were obtained and processed shipboard. Whole round samples for DNA-based studies were capped with ethanol-sterilized endcaps, transferred to the microbiology laboratory, and stored briefly at 4°C in heat-sealed tri-foil gas-tight laminated bags flushed with nitrogen until processing. Masks, gloves and laboratory coats were worn during sample handling in the laboratory where core samples were transferred from their gas-tight bags onto sterilized foil on the bench surface inside a Table KOACH T 500-F system, which creates an ISO Class I clean air environment (Koken Ltd., Japan). In addition, the bench surface was targeted with a fanless ionizer (Winstat BF2MA, Shishido Electrostatic Co., Ltd., Japan). Within this clean space, the exterior 2 cm of the extruded core section were removed using a sterilized ceramic knife. The core interior was transferred to sterile 50-mL Falcon tubes, labeled, and immediately frozen at -80°C for post cruise analyses. For RNA-based studies, sampling occurred immediately after core retrieval on the core receiving platform by sub-coring with a sterile, cutoff 50cc syringe into the center of each freshly cut core section targeted. These sub-cores were immediately frozen in liquid nitrogen and stored at -80°C. On board data collection was performed by the IODP385 scientists and the technical stuff participating on the Expedition.
Timing and spatial scale	Sediment cores were collected during a sampling expedition that took place in Guaymas Basin between Sept. 15 to Nov. 15, 2019.
Data exclusions	No data were excluded from the study
Reproducibility	No sediment treatments or experiments were performed in this study.
Randomization	Sampling strategy followed the in situ temperature and depth measurements/profiles, and did not involve randomized sampling or trials
Blinding	No randomized trials or treatments were performed on this study

Did the study involve field work? ☒ Yes ☐ No

Field work, collection and transport

Field conditions	The IODP385 expedition took place in Guaymas Basin from September 16 to November 16 2019. Temperature ranged from 22.6 to 31°C with occasional rainfalls.
Location	Sampling location was Guaymas Basin, Mexico. Elevation: 29 meters. Sampling Locations: 27°38.2325′N, 111°53.3406′W; 27°30.4561′N, 111°40.6980′W; 27°28.3317′N, 111°28.7844′W; 27°15.1602′N, 111°30.4163′W; 27°12.3887′N, 111°13.1943′W; 27°33.2906′N, 111°32.9665′W.<br>Water depth ranged from 1586.1 to 2000.8 meters below sea surface.

Access & import/export	The research expedition in Guaymas Basin was performed with Deep-sea drill ship JOIDES Resolution. We verify that all samples were collected in a responsible manner and in compliance with local, national, and international laws.
Disturbance	No disturbance was caused by the study. Overall, the IODP385 Expedition was performed in a responsible manner and in compliance with local, national, and international laws.

Reporting for specific materials, systems and methods

We require information from authors about some types of materials, experimental systems and methods used in many studies. Here, indicate whether each material, system or method listed is relevant to your study. If you are not sure if a list item applies to your research, read the appropriate section before selecting a response.

Materials & experimental systems

n/a	Involved in the study
☒	☐ Antibodies
☒	☐ Eukaryotic cell lines
☒	☐ Palaeontology and archaeology
☒	☐ Animals and other organisms
☒	☐ Clinical data
☒	☐ Dual use research of concern
☒	☐ Plants

Methods

n/a	Involved in the study
☒	☐ ChIP-seq
☒	☐ Flow cytometry
☒	☐ MRI-based neuroimaging

Plants

Seed stocks	N/A
Novel plant genotypes	N/A
Authentication	N/A
